# Effects of the Humorous Characteristics of the School Principals on School Health

**DOI:** 10.3389/fpsyg.2021.628775

**Published:** 2021-02-24

**Authors:** Mehtap İnceler, Ahmet Güneyli

**Affiliations:** ^1^Education Faculty, Near East University, Nicosia, Cyprus; ^2^Education Faculty, European University of Lefke, Nicosia, Cyprus

**Keywords:** emotions, emotional management, humor, organizational health, school principal, school management

## Abstract

In this study, teachers' views are evaluated to determine whether there is a relationship between the humor styles of school principals and the health of an organization. The study is based on a mixed research approach with both quantitative and qualitative aspects. In the quantitative study, the teachers were asked to describe their principals' senses of humor using the “humor behavior scale” and to evaluate the organization in which they worked using the “organizational health inventory.” In the qualitative dimension, principals working in primary schools were interviewed and asked to evaluate their own sense of humor and the school's health. When the quantitative findings were examined, the regression results showed that school principals with a cynical style of humor negatively predicts organizational health, and productive social and affirmative styles of humor positively predict organizational health. In the qualitative study, the content analysis results revealed that a school administrator who uses humor effectively is capable of effectively solving problems in the school. However, it has been stated that the humor style of the school principal can have negative effects as well as positive effects. School principals are advised to recognize the teachers with whom they work and to distinguish which teachers are receptive to humor.

## Introduction

In this study, emotional intelligence in school administration and the humor characteristics of school administrators—which is a component of emotional intelligence in education—were examined. The starting point of this paper is to display whether the humor characteristics of school administrators affect school health. In a developing territory like Northern Cyprus, some chronic problems related to education are occurring (Erden and Erden, 2019). For example, in terms of teacher-related problems, teachers have been found to have insufficient internal motivation, lack job satisfaction, and have little desire to improve themselves, and, as a result, experience burnout (Özberk, 2015; Emiroglu, 2017; Akartaş, 2018). In addition to teachers, students also experience some serious problems in the education process (e.g., absenteeism, social adaptation problems, academic failure, etc.; Mutluoglu and Bulut-Serin, 2010; Davutoglu, 2011). The concept of “school health” comes to the fore when problems originating from teachers and students are evaluated in terms of the education system of Northern Cyprus. When we consider why schools are not adequately healthy, we are led to consider the profiles of school administrators. There are basic problems arising from the education system, such as not having leadership qualities, ineffective management skills, and the lack of qualified management training. It is also worth noting that negative personal characteristics of school administrators also play a role (Eray, 2015; Serin-Tanyel and Görkem, 2019; Yorulmaz, 2020). Some studies state that students are reluctant to communicate with the school administrator and generally have a negative attitude toward the school administrator (Yerel, 2013; Üstün-Aksoy, 2018). Teachers also state that administrators are mostly strict, non-communicative, and very formal—in short, school administrators are not open to compromise (Kondoz, 2007). Based on these findings, it is clear that the humor characteristics of school administrators can affect both teachers and students. For this reason, we investigate the extent to which a school administrator's sense of humor affects school health.

In the literature, humor characteristics of school administrators (Hurren, 2006; Yilmaz, 2011; Otrar and Findikli, 2014; Yirci et al., 2016; Çelikten and Çelikten, 2018; Çetin and Altun, 2018; Dinç and Cemaloglu, 2018; Sahin, 2018; Ahmad and Bakhsh, 2019; Bakhsh et al., 2019) and the organizational health of schools (Hoy and Woolfolk, 1993; Gürsel, 1998; Tsui and Cheng, 1999; Akil, 2005; Celep and Mete, 2005; Korkmaz, 2005; Türker, 2010; Lenka and Kant, 2017; Parlar and Cansoy, 2017; Özgenel and Aksu, 2020) are explored separately. Thus, very few studies have examined administrator humor and school health in relation to each other. However, a study conducted in Turkey (Recepoglu, 2011) did explore this correlation. According to the findings, the most important factor that can affect the health of the school is the behavior of the school principal as a teaching leader, and the role of the school principal is more influential than other factors. Therefore, we expect that humor, which is considered an effective leadership characteristic, will contribute positively to school principals creating a healthy school. Furthermore, Özdemir and Recepoǧlu (2010) also found a significant and positive relationship between school principals' humor styles and the health of schools. Organizational health scores of teachers working with school principals who have a distinctively productive humor style were at the highest level, while those of teachers working with school principals with a non-humorous style had the lowest organizational health scores. The results of the aforementioned study clearly reveal that the humor styles of school principals have important effects in determining the organizational health of schools. Based on the results of this study, it will be possible to evaluate whether the relationship between the humor styles of school principals and the organizational health of schools differs after 10 years.

When evaluating recent literature, we found that current studies frequently attempt to assess the relationship between the humor characteristics of school administrators and school climate (Matthias, 2014; Çanak and Coşkun-Demirpolat, 2016). However, in the present work, the relationship between administrators' humor style and the organizational health of schools, which is a less emphasized and researched subject, is discussed. In this way, we aim to contribute to the literature by obtaining up-to-date data on the organizational health of schools and the personal characteristics of school administrators in a developing territory, such as Northern Cyprus.

## Conceptual Framework

In this section, three basic concepts are explained: the importance of emotional management in education, humor and its functions in school management, and the organizational health of schools.

### Importance of Emotional Management in Education

According to many social scientists, to try understand human nature by isolating it from the power of emotions is not a rational approach. Social psychologists believe that emotions guide many situations, such as danger, painful loss, and progress toward a goal by enduring difficulties. Every emotion prompts people to act in one way or another and directs them to cope with difficulties (Ekman, 1992). Emotions allow people to face uncertainty, set long-term goals, choose among various alternatives, make predictions about the future, deal with the unknown, and make quick decisions (Damasio, 1994). Emotions are important in understanding the individual, as well as their behaviors and thoughts (Titrek, 2013).

Individuals should have emotional intelligence to establish effective communication and be solution-oriented, as communication skills and emotional intelligence have positive impacts on the individual as well as others with whom they communicate. These two concepts, which are important in terms of the effectiveness and efficiency of living or working together, are closely related (Avci, 2019). Titrek (2013) defines emotional intelligence as the correct expression of the emotions of oneself and others, professional reflection on these emotions in social relations, the selection of these emotions, and explaining the information obtained in the form of an opinion.

The ability to manage emotions prevents a person from being a prisoner to their feelings. Managers, who are responsible for organizational effectiveness, should have some qualifications in effective leadership. Leaders who are able to manage their emotions find ways to manage and benefit from their emotional impulses; however, like other people, they are also affected by them. Leaders with such qualifications can act calmly in difficult times, when the atmosphere becomes heavy, and in times of crisis (Aysel, 2006). Demir (2010) underlined that effective employment of emotional intelligence directs the organization toward positive thinking, even under negative working conditions, and states that it is through this process that motivation is ensured, and organizational conflicts are minimized. Karadavut and Çetin (2018) state that emotional intelligence in organizations is of critical importance for leaders. Effective leadership requires awareness of other people's emotions and the ability to manage emotions. Studies show that emotional intelligence is closely related to leader effectiveness. Specifically, the higher the leadership characteristic, the more effectively the emotional intelligence is used (Dulewicz and Higgs, 2003; Goleman, 2005; Titrek et al., 2014). Aysel (2006) states that the high motivation required in organizations cannot be achieved only with material elements because emotions are also important. Flam (1990) observed that emotions in organizations affect all members and the functioning of the organization, and serve the unit's vision by creating a common culture.

It is known that manager behaviors are very important in education organizations where interpersonal relations are extremely critical. Manager behaviors can directly affect the emotions of teachers and students and improve or hamper their performance. An education administrator who is aware of what is expected of them should carefully choose the behaviors they display (Erçetin, 2000). Humor is one of the behavioral characteristics that school administrators should carefully assess. The school administrator can use humor to effectively manage emotions in the school, thus affecting the thoughts, feelings, and actions of teachers and students.

### Humor as an Emotion and Its Functions in School Management

Humor can be understood as a way to express feelings and thoughts. The main purpose of humor is to make you laugh, but apart from that, it also reveals social problems and deficiencies and helps to correct them. A sense of humor, which is regarded as a personality trait, includes individuals' perceptions, bearing, and the practice of humor (Özdogru, 2018). Based on this, we understand that humor is an emotion.

Groups in workplaces and businesses are influenced by humor since it is a main component of human relationships that is shared by all people. Humor has the ability to make workplaces more pleasant by relaxing the weighty environment. In other words, people can share their knowledge and opinions more plainly, and relationships will flourish in pleasant environments created by humor. However, managers often ignore the advantages of humor. People like to perceive humor, but they are often not comfortable in showing their contentment when they experience it in a work setting (Mathew and Vijayalaksmi, 2017).

Furthermore, humor is one of the easiest and most effective ways of improving communication. This non-threatening form of communication can have lasting benefits for both principals and teachers (Vickers, 2004). Individuals who have a strong sense of humor and use humor more frequently are said to have better understanding and intuition than others (Booth-Butterfield and Booth-Butterfield, 1991). Humor in workplaces contributes to motivating staff, making their interpersonal communication more powerful, and reducing disagreements when it is used successfully. It is not only for enjoyment but also serves as a multifunctional tool in business. It is the manager's responsibility to decide on the humor style most suitable for the organization by taking into consideration their personal characteristics to reach the intended results (Alan and Sen, 2016). People are affected by their emotions because these feelings significantly affect their concentration, productivity, inclination, benefits obtained, and ability to judge information. People transmit their emotions to others through distinct tones and bodily gestures. Therefore, it could be stated that emotions can serve as an effective tool for management and collaborative group work in the field of business (Fisher, 2019).

The effects of successful emotion management in terms of individual and managerial aspects are very important for organizations to achieve a healthy work environment. From an individual perspective, emotional problems are one of the most common issues faced by employees working in today's organizations. Other challenges include routinization, dismissal, inertia (stagnation), feelings of burnout, insecurity, loss of performance, future anxiety, competition, and selfishness (Töremen and Çankaya, 2008). Especially in today's organizations, the increasing feelings of psychological violence, stress, depression, and burnout are fueled by the neglect and abuse of basic human emotions (Töremen and Çankaya, 2008). For this reason, a manager who can cope with negative emotions and use humor contributes to the health of the organization. This is particularly important in human-oriented organizations that provide services, such as educational institutions. As the general characteristics and organizational climate of organizations have a direct effect on individuals, individual and shared feelings can also directly affect organizational climate (Langelier, 2006).

According to recent studies of emotion management, humor is seen as a social tool serving the maintenance of interpersonal relationships. To control and direct the emotions of staff, humor is often used. It has also been applied to enhance and improve the emotional pattern of a specific environment, thus reducing the risk of external peril by providing common satisfaction for people sharing a workspace (Francis, 1994). For harmonious teamwork in organizations, humor contributes to eliminating the discomfort of individuals and replaces standard relations with more efficient ones (Töremen and Çankaya, 2008). Based on this principle, we concluded that humor is an important variable in the emotional dimension of the management of educational institutions.

Laughter and humor are indispensable components of a healthy and long life. If we do not include humor in our lives, the ability to lead a physiologically and psychologically healthy life would be very challenging. McDoughall (cited by Hurren, 2001) stated that laughter and humor are critical values. He said that humor makes us laugh, and the act of laughing accelerates the respiratory and circulatory processes. The brain is thus stimulated by increased blood flow and activated by greater mental power. Research shows that humor has a positive effect on health in both physiological and psychological terms and that it helps us to cope with stress, which is one of the greatest health problems of the modern age. Thus, it is possible to comprehend that humor will have a positive effect on both individual health and the organizational health of schools. Moreover, it is evident that humor is of great importance in our daily lives as well as in our education.

Assuming that managers are one of the most important factors in the formation of organizational culture and climate, it can be beneficial to learn the humor styles of school principals in practice to determine the prerequisites on which common organizational culture and climate are based. Considering that humor is not always beneficial, school administrators should be investigated in detail.

### Organizational Health of Schools

The organizational health of educational institutions refers to an organization not being content with its current operational environment but continues to develop and improve its coping and living strategies. The concept of organizational health, which emphasizes organizational efficiency and the growth and development of the organization, is very critical for all organizations. Schools are the fundamental institutions of education systems; therefore, increasing the effectiveness and success of schools and sustaining their development is possible by establishing a healthy structure. Considering that schools raise young people in society and prepare healthy individuals for the future, it is important for both educators and students for schools to be healthy organizations. Healthy individuals are raised only in healthy school environments. Therefore, it is necessary to create a healthy school for personal, social, and academic learning (Akbaba-Altun, 2001; Aksulu-Köse and Güçlü, 2018). Studies show that organizational health positively affects student achievement (Korkmaz, 2005; Henderson, 2007; Mirzajani and Morad, 2015) and organizational health increases school effectiveness (Hoy et al., 1990).

Healthy schools represent ideal work environments, where employees respect, love, and help each other. Teachers working in healthy schools are more productive, administrators are more considerate, and students are more successful. In terms of educational institutions, organizational health is a good indicator of the psychosocial status of a school. The purpose of determining the organizational health of schools is to identify the factors that are effective in making the school healthy or unhealthy. School principals who know whether their schools are healthy or not can direct their behaviors according to these results by obtaining information about their schools (Hoy et al., 1991).

School principals are one of the most important elements that affect the organizational health of schools. It can be argued that the impact of the principal, the commitment of teachers, the richness of sources and materials at the school, and academic attention have positive impacts on the organizational health of schools. As the impact of the principal increases, teacher impact, source support, and academic attention also improve (Yarim and Korkmaz, 2019). For example, thanks to the leadership behaviors exhibited by school principals, teacher–student motivation and success can be increased, thus ensuring the health of the school as an organization (Cemaloglu, 2007; Aksulu-Köse and Güçlü, 2018).

Although a healthy organizational structure is a desirable situation, it does not always exist, and some organizational behaviors can negatively affect the health of an organization (Tabak et al., 2018). In a study conducted by Tabak et al. (2018) on organizational health, the authors concluded that “workaholism” is a variable that negatively affects organizational health, and teachers with high levels of addiction to work have a low perception of organizational health. Another variable that negatively affects organizational health is work stress: organizational health may be low in organizations with high work stress (Gül, 2007).

## Aim and Importance of the Study

When the explanations provided by the literature are examined, the necessity and positive effects of humor in daily life and organizational environments are clear. The successful use of humor creates positive effects within an organization, whereas unconscious and careless humor inevitably affects the health of the organization negatively. For this reason, the frequency of humor and the way it is used by school administrators are important for organizational health. Studies show that the humorous behaviors of principals affect their communication with teachers and, thus, the health of the school. For this reason, it is necessary to determine the humor styles of principals according to the teachers' perceptions, to question the health of the organization from the perspective of the teachers, and to identify the relationship between these two variables. Researching this issue may reveal important findings that can be beneficial for improving the organizational health of schools and, therefore, the quality of the education provided. As this study represents the first research of its kind in Cyprus, and as it is research aimed exclusively at primary schools, this will allow us to evaluate school principals in terms of humor, not only according to the perceptions of teachers, but also by including the self-evaluations of the administrators.

In this study, the research question is: What is the effect of primary school principals' humor styles on the organizational health of the schools? To answer this question, we explored the following sub-questions.

*Quantitative dimension:* According to the opinions of teachers participating in the research:

- What is the distribution of the scale scores of school administrators from evaluating humor behaviors and the scores obtained from the organizational health inventory?
- Is there a relationship between the scale scores of school administrators from evaluating humor behaviors and the scores obtained from the organizational health inventory?
- To what extent do the humor behaviors of school administrators predict organizational health?

*Qualitative dimension:* How do school administrators' humor styles affect the health of the organization in which they work?

## Methods and Materials

The aim of this study is to investigate the relationship between school principals' humor styles and organizational health, according to teachers' perceptions. In addition, this study also asks the school principals to evaluate themselves.

### Research Model

In the quantitative dimension of this study, a relational survey model was used to examine the effects of the humor behaviors of school principals (measured by the opinions of the primary teachers) on organizational health. The qualitative section, on the other hand, was based on a case-study model. The combination of qualitative and quantitative data in this study can be explained as follows: While we aimed for teachers to evaluate the humor characteristics of school administrators and the organizational health of schools, we also asked school administrators working in the same schools to evaluate themselves. Thus, data diversification was conducted by obtaining *quantitative* data from teachers and *qualitative* data from school administrators on humor and school health issues. We aimed to study the subject comprehensively by obtaining both quantitative and qualitative data on the basis of the same research subject (humor and school health). In addition, the research was not limited to the attitudes of the teachers, but also included that of school administrators, so the subject was approached from two perspectives.

### Study Group

The universe of the quantitative study consisted of teachers who were working at public primary schools of the North Cyprus Ministry of National Education in the 2017–2018 academic year. According to the Turkish Cypriot Teachers Union (KTÖS), the number of teachers working in public primary schools affiliated with the Department of Primary Education was 1,482. Since it was challenging to reach the entire study universe based on time and cost constraints, a sample was selected using stratified random sampling. Teachers in the research universe were stratified to the districts in which they worked (Nicosia, Famagusta, Kyrenia, Güzelyurt, and Iskele), and the sample was selected using simple random sampling (Table 1). Accordingly, it was found that the number of people who should be interviewed was 283 with a 95% confidence interval and 5.2% sampling error.

**Table 1:** Distribution of teacher numbers by regions.

**Region**	**Number of teachers**	**Ni/N**	**Number of samples**
Nicosia	477	0.32	91
Famagusta	401	0.27	76
Kyrenia	277	0.19	54
Güzelyurt	184	0.12	34
Iskele	143	0.10	28
Total	1482	1.00	283

In the qualitative approach, purposive sampling was used, and school administrators were selected in accordance with convenience sampling. Within the scope of the quantitative research, data were obtained from the teachers at the schools studied. In the qualitative segment, school administrators working in the same schools were reached. Thus, data were obtained from both teachers and school administrators in the schools covered by the study.

As seen in Table 2, 17 school administrators were interviewed in the qualitative dimension of the study. The demographic and background characteristics of these administrators are presented in Table 2.

**Table 2:** Distribution of the demographic characteristics of school principals.

	**Number (*n*)**	**Percentage (%)**
**Gender**		
Female	6	35.29
Male	11	64.71
**Age**		
31–40 years of age	2	11.77
41–50 years of age	10	58.82
51 years and older	5	29.41
**Education level**		
Undergraduate	11	64.71
Graduate	6	35.29
**Professional seniority**		
10 years and under	2	11.77
11–20 years	10	58.82
21 years and over	5	29.41
**Tenure in principal position**		
1–5 years	4	23.53
6–10 years	7	41.17
11–20 years	4	23.53
21 years and over	2	11.77

### Data Collection Tools

A four-part questionnaire was used to collect the data. The first part of the questionnaire contained socio-demographic questions, the second part included the humor behaviors scale, the third part contained the organizational health inventory, and the fourth part was the interview form.

#### Socio-Demographic Questions

In this form, which was developed by the researchers, questions about descriptive characteristics, including age, gender, educational status, seniority, term of duty at the current school, and branch of the teachers participating in the research, were included.

#### Humor Behaviors Scale

To determine the opinions of teachers regarding the humor behaviors of school principals, the humor behaviors scale developed by Cemaloglu et al. (2012) was used. The humor behaviors scale consists of 30 positive and negative suggestions developed in a Likert-type format using a five-point rating. The responses to the propositions in the scale are scored as: strongly disagree = 1 point, disagree = 2 points, moderately agree = 3 points, agree = 4 points, and strongly agree = 5 points. The factor structure of the scale was investigated using AMOS 21.0.0. A five-factor model was hypothesized, and the results confirmed the predicted factor structure. When the model fit was investigated with the commonly accepted values of the fit indices, it was observed that the model had a good fit with the data. The scale shows that there are five factors, namely, cynical humor style, productive social humor style, affirmative humor style, rejective humor style, and non-humor style. These five factors explain 70.1% of the variance of the entire scale. There is no general total score of the scale, and a high score obtained from the sub-dimensions indicates that the humor behaviors associated with the related sub-dimensions were developed. When the findings of the reliability study of the scale were examined, a Cronbach's alpha value of 0.919 was found, suggesting a good fit. When the reliability analysis of the subscales was conducted, Cronbach's alpha was 0.943 for cynical humor, 0.923 for productive social humor, 0.864 for affirmative humor, 0.855 for rejective humor, and 0.895 for non-used humor (Cemaloglu et al., 2012). For the reliability of the data collected in this study, the Cronbach's alpha value for the full scale was 0.789. These findings suggest that the scales are accurate indicators of the concepts being measured.

#### Organizational Health Inventory

To determine the opinions of the teachers on the organizational health of their school, the organizational health inventory, which was originally developed by Hoy and Miskel (1991) and adapted to Turkish by Cemaloglu (2006), was used. The organizational health inventory consists of 44 positive and negative questions prepared using a quadratic rating. Positive propositions included in the scale are randomly = 1 point, occasionally = 2 points, often = 3 points, and always = 4 points. Negative propositions are scored as: randomly = 4 points, occasionally = 3 points, often = 2 points, and always = 1 point. The validity and reliability study conducted revealed that there are seven sub-dimensions that explain 74% of the total variance in the scale. The Corporate Integrity sub-dimension consists of items numbered 1, 8, 15, 22, 29, 36, and 39; the Impact of the Principal sub-dimension consists of items 2, 9, 16, 23, and 30; the Courtesy sub-dimension consists of items 3, 10, 17, 24, and 31; the Incentive Structure sub-dimension consists of items 4, 11, 18, 25, and 32; the Resource Support sub-dimension consists of items 5, 12, 19, 26, and 33; the Morale sub-dimension consists of items 6, 13, 20, 27, 34, 37, 40, 42, and 44; and the Academic Emphasis sub-dimension consists of items numbered 7, 14, 21, 28, 35, 38, 41, and 43. A high score obtained from the relevant sub-dimension indicates that the organizational health of that sub-dimension is high. The Cronbach's alpha coefficient for the overall scale was 0.91 (cited in Recepoglu, 2011). In this study, the Cronbach's alpha internal consistency coefficient for the overall scale was 0.946, suggesting a reliable measure.

#### Interview Form

The interview form prepared for the qualitative part of the study consisted of four open-ended questions and two sections. In the first section, we aimed to reveal the impact of humor on school management and to determine how school administrators evaluated their humor characteristics. In the second section, the purpose was to ensure that school administrators provided their opinions on the impact of humor on the organizational health of the school. Attention was paid to ensure that the questions were as clear as possible and easy to understand, and that they provided explanations and detailed answers. We also designed them so that they were not multidimensional to avoid creating an unnecessary question burden on the interviewees. While preparing the questions, three academics working in the fields of educational administration, educational psychology, and educational sociology were consulted about the scope and appropriateness of the questions. Before applying the interview questions, a pilot study was performed; two school administrators were interviewed, and the questions were finalized based on the results.

### Procedure

The research was conducted during the 2017–2018 academic year. Before sharing the scale and the opinion form, permission was obtained from the Scientific Ethics Committee of Near East University and the Ministry of Education of Northern Cyprus. Before administering the scales to the teachers, verbal permission was obtained from the school principals. The questionnaires were administered by the researchers during school working hours. The teachers completed the scales confidentially. The interviews, on the other hand, were planned according to appointments made by the school principals and were conducted in the schools by the researchers during or just after working hours. The interviews were recorded on a tape recorder with the permission of the school principals. School principals who did not agree to have their voice recorded were asked to answer the questions in writing, or their answers were written down by the researcher.

### Data Analysis

IBM SPSS 24.0 was used for the statistical analysis of the data collected through the questionnaire. Frequency analysis was used to determine the distribution of teachers according to their background characteristics. Descriptive statistics of the scores obtained from the humor behavior scale and organizational health scale are provided. The normal distribution status of the scale scores was examined using the Kolmogorov–Smirnov test, the QQ graph, and skewness–kurtosis coefficients. The data were found to conform to a normal distribution. The results of the Pearson correlation analysis were used to determine the relationships between the scores of the teachers' humor behaviors scale and the organizational health inventory. Structural equation modeling was employed using AMOS 21.0.0 and was used to examine the predictive strength of the scores obtained by the teachers from the Humor Behavior Scale as per the scores obtained from the organizational health scale. To further examine the independent contributions of cynical humor style, productive social humor style, affirmative humor style, rejective humor style, and non-humorous style in predicting organizational health, a simultaneous regression analysis was conducted using the organizational health inventory score as the dependent variable.

Model fit was investigated using the chi-square goodness-of-fit test (a lower chi-square value indicates a better fit; see Loehlin, 1998), the root mean square error of approximation (RMSEA; a better fit is found when RMSEA ≤ 0.06; see Steiger, 1990), the standardized root mean square residual (SRMR; better fit indicated by SRMR ≤ 0.08; see Chen et al., 2008), the Tucker–Lewis index (TLI; a better fit exists when the obtained value is between 0 and 1; see Tucker and Lewis, 1973), and the comparative fit index (CFI; a better fit is noted when CFI ≥ 0.95; see Bentler, 1990).

Data obtained in the qualitative dimension of the study were analyzed using content analysis. The following stages were applied:

- The responses of the school administrators to the interview questions were written without any changes.
- Each of the interview questions was evaluated as a separate category.
- Views expressed by school administrators in each category were analyzed, keywords were determined, and codes were created.
- The determined codes were listed, similar codes were grouped and classified, and themes were created.
- Themes were presented in tables.
- To explain the themes, direct quotations were provided that express the views of the school administrators who participated in the study.

To ensure validity in the qualitative data analysis, the data were written down in detail, and the process of reaching the findings was explained clearly. The responses by the interviewed managers were frequently included through direct quotations, and the findings of the research were explained based on these quotations. For reliability, two researchers coded the data separately, and then the consistency in the analyses of the two researchers was examined. In the consistency analysis conducted on one question, the two researchers reached similar themes from the same data. In the following process, during the data analysis, the two researchers worked together each time, eliminating the differences of opinion and performing the data analysis.

## Results

### Results on the Quantitative Dimension of the Study

Descriptive statistics including mean, standard deviation, and lower and upper values of the scores obtained from the teachers' humor behavior scale and organizational health inventory are given in Table 3. The results indicate that the teachers who participated in the study received $\bar{x}$ = 1.69 ± 0.78 points from the cynical humor style sub-dimension, $\bar{x}$ = 2.75 ± 0.85 points from the productive social humor style sub-dimension, $\bar{x}$ = 3.40 ± 0.86 points from the affirmative humor style sub-dimension, $\bar{x}$ = 1.87 ± 0.82 points from the rejective humor style sub-dimension, and $\bar{x}$ = 1.87 ± 0.82 points from the non-humorous style sub-dimension.

**Table 3:** Scores of teachers from the humor behavior scale and organizational health inventory (n = 283).

	***n***	**$\bar{x}$**	***s***	**Min**	**Max**
Cynical humor style	283	1.69	0.78	1.00	4.63
Productive social humor style	283	2.75	0.85	1.00	5.00
Affirmative humor style	283	3.40	0.86	1.00	5.00
Rejective humor style	283	1.87	0.82	1.00	4.60
Non-humorous style	283	2.10	1.06	1.00	5.00
Corporate integrity	283	2.88	0.50	1.00	4.00
Impact of the principal	283	2.80	0.54	1.20	4.00
Courtesy	283	2.89	0.67	1.20	4.00
Incentive structure	283	3.04	0.68	1.00	4.00
Resource support	283	2.80	0.70	1.00	4.00
Morale	283	2.93	0.54	1.56	4.00
Academic emphasis	283	2.74	0.54	1.25	4.00

Regarding the organizational health inventory, the teachers received $\bar{x}$ = 2.88 ± 0.50 points from the corporate integrity sub-dimension, $\bar{x}$ = 2.80 ± 0.54 points from the impact of the principal sub-dimension, *x* = 2.89 ± 0.67 points from the courtesy sub-dimension, $\bar{x}$ = 3.04 ± 0.68 points from the incentive structure sub-dimension, $\bar{x}$ = 2.80 ± 0.70 points from the resource support sub-dimension, $\bar{x}$ = 2.93 ± 0.54 points from the morale sub-dimension, and $\bar{x}$ = 2.74 ± 0.54 points from the academic emphasis sub-dimension.

Table 4 shows the results of the Pearson correlation analysis to determine the relationships between the scores obtained by the teachers from the humor behavior scale and the organizational health inventory. The scores obtained from the teachers for the sub-dimensions of cynical humor style, rejective humor style, and non-humor style, and the scores obtained from the sub-dimensions of corporate integrity, effect of the principal, courtesy, incentive structure, resource support, morale, and academic emphasis included in the health inventory, show statistically significant and negative correlations (*p* < 0.05). As the scores obtained by teachers from the cynical humor style, rejective humor style, and non-humor style sub-dimensions on the behaviors scale increase, the scores for corporate integrity, impact of the principal, courtesy, incentive structure, resource support, morale, and academic emphasis on the health inventory decrease.

**Table 4:** Correlations between the scores of the teachers' humor behavior scale and organizational health inventory (*n* = 283).

		**Cynical humor style**	**Productive social humor style**	**Affirmative humor style**	**Rejective humor style**	**Non-humorous style**
Corporate integrity	*r*	−0.340	0.089	0.246	−0.404	−0.254
	*p*	0.000^*^	0.137	0.000^*^	0.000^*^	0.000^*^
Impact of the principal	*r*	−0.268	0.322	0.435	−0.333	−0.315
	*p*	0.000^*^	0.000^*^	0.000^*^	0.000^*^	0.000^*^
Courtesy	*r*	−0.422	0.502	0.590	−0.499	−0.558
	*p*	0.000^*^	0.000^*^	0.000^*^	0.000^*^	0.000^*^
Incentive structure	*r*	−0.325	0.341	0.455	−0.340	−0.369
	*p*	0.000^*^	0.000^*^	0.000^*^	0.000^*^	0.000^*^
Resource support	*r*	−0.241	0.277	0.443	−0.293	−0.273
	*p*	0.000^*^	0.000^*^	0.000^*^	0.000^*^	0.000^*^
Morale	*r*	−0.313	0.203	0.300	−0.349	−0.196
	*p*	0.000^*^	0.001^*^	0.000^*^	0.000^*^	0.001^*^
Academic emphasis	*r*	−0.330	0.332	0.463	−0.288	−0.299
	*p*	0.000^*^	0.000^*^	0.000^*^	0.000^*^	0.000^*^

* *p < 0.05*.

The scores obtained for the teachers for the social humor and affirmative humor sub-dimensions in the humor behaviors scale and the scores obtained from the sub-dimensions of corporate integrity, impact of the principal, courtesy, incentive structure, resource support, morale, and academic emphasis in the health inventory, reveal statistically significant correlations (*p* < 0.05). These correlations are positive, and as the scores for the former group increase, those for the latter group increase as well.

**Figure 1:**
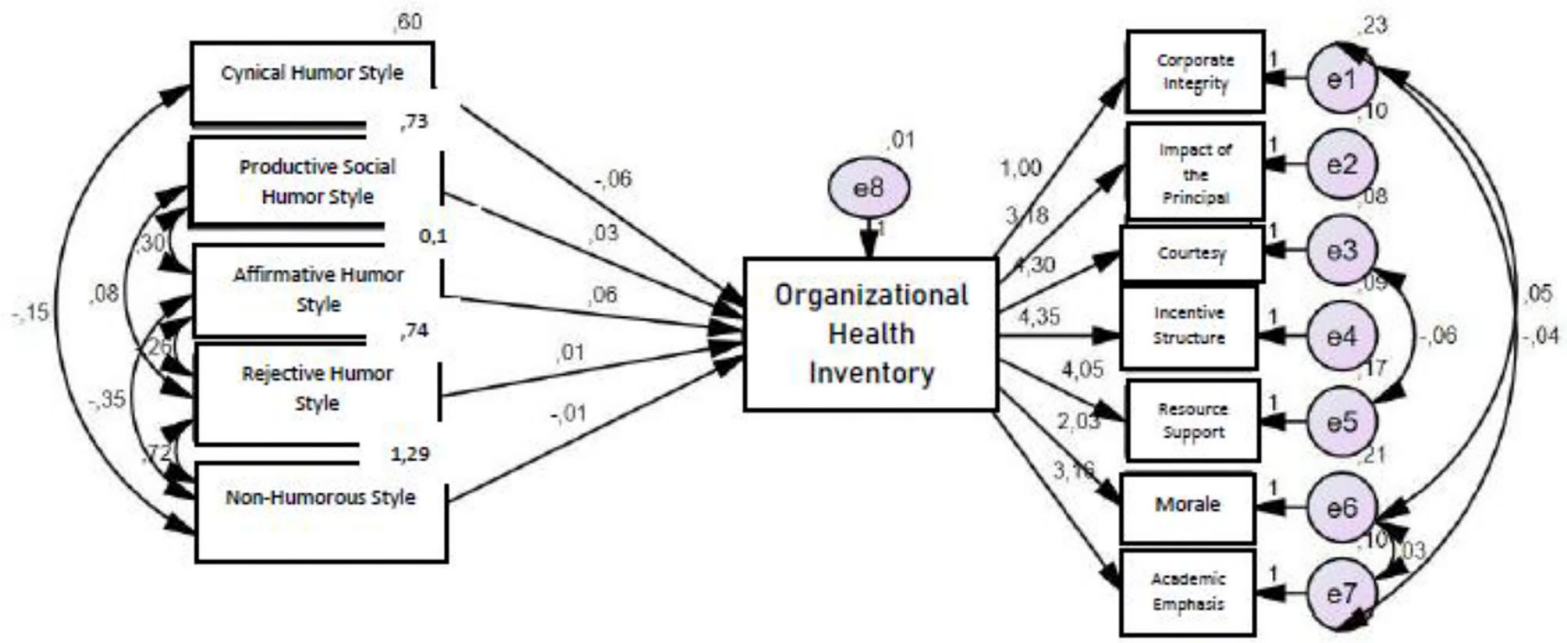
Path analysis of the effect of teachers' views on school principals' humor behaviors on organizational health. The covariance between the error terms is caused by the modification indices specified by the software to increase the goodness-of-fit values of the model.

Structural equation modeling was used to examine the effect of the teachers' opinions of school principals' humor behaviors on organizational health. Figure 1 shows that the scores obtained for cynical humor style, productive social humor style, affirmative humor style, rejective humor style, and non-humorous style predict the organizational health inventory scores; the findings reveal that the CFI, NFI, GFI, and AGFI values of the model are 0.913, 0.935, 0.926, and 0.907, respectively. Additionally, the X^2^/sd value of the model was found to be 3.645, and the RMSEA value was 0.781. According to these results, the model was found to have a good fit.

Table 5 indicates that the scores obtained from the cynical humor style, productive social humor style, and affirmative humor style significantly predict the organizational health inventory scores (*p* < 0.05). The cynical humor style subscale scores are negatively related to the organizational health inventory scores, whereas the relationships between productive social humor style and affirmative humor style subscales and the health inventory are positive. The scores obtained from the sub-dimensions of rejective humor and non-humor style did not significantly predict the scores obtained from the organizational health inventory (*p* > 0.05).

**Table 5:** Regression results on the effect of teachers' views on the humor behaviors of school principals on organizational health.

	**Estimation**	**S.E**.	**C.R**.	***p***
Cynical humor style	−0.055	0.015	−3.636	0.000^*^
Productive social humor style	0.033	0.012	2.789	0.005^*^
Affirmative humor style	0.055	0.017	3.337	0.000^*^
Rejective humor style	0.006	0.012	0.469	0.639
Non-humorous style	−0.012	0.009	−1.293	0.196

* *p < 0.05*.

### Results on the Qualitative Dimension of the Study

In light of the qualitative data obtained from primary school principals, the following themes have been developed:

**Theme 1: A school principal who uses humor effectively can cope with problems**.

The school administrators who were interviewed in this study stated that they could solve problems when they used humor effectively. Under this theme, three sub-themes were formed, namely, “it is effective in tense situations,” “it has a calming and moderating effect,” and “it ensures that teachers assume different viewpoints to situations.” Two school principals who stated that it was effective in tense situations reported that “it reduced stress in stressful situations” (SP13) and “it relieved tense environments” (SP12). Four of the school principals who indicated that “it has a calming and moderating effect” revealed their opinions as follows:

- It creates an intimate environment that gives confidence in solving problems. (SP10)
- It puts a smile on people's faces. (SP5)
- It relaxes the environment by creating the feeling that the problem is not too serious. (SP17)
- The humorous approach works because it allows the message to be transmitted to the other party more smoothly so they can solve the problem. (SP12)

Participants who stated that the school principal's humor style enabled teachers to think and comment differently made the following statements:

- It prevents individuals from saying “no.”
- It prepares the ground for the formation of positive thoughts.
- It allows you to look at events from different perspectives.

**Theme 2: The effect of humor on school health can be both positive and negative**.

One school administrator (SP16) stated that humor adversely affected school health: “I refrain from humor because I think it will adversely affect and undermine school order and discipline.” Contrary to this view, some school administrators stated that humor positively affected school health. Two of these views are presented below:

- I have repeatedly observed that it affects the school atmosphere positively. (SP14)
- Humor certainly has a positive effect on the school environment. (SP13)

## Discussion

### Discussion on the Quantitative Dimension of the Study

#### Descriptive Results

In this study, when the findings of the teachers' evaluations of the humor behaviors of school administrators are examined (see Table 2), the average scores of the negative humor style dimensions (cynical and rejective) were at their lowest level. On the other hand, the arithmetic mean value was found to be moderate in positive humor style dimensions (affirmative and productive social). Based on these findings, it can be said that teachers are not negatively affected by school administrators' humor behaviors. However, school administrators do not always use humor in a manner that affects teachers positively. It may be important for the school administration to avoid negative perceptions of the principal's humor behavior to avoid conflict between teachers and to maintain healthy communication. In this study, a close-to-average value in the non-humorous style dimension was obtained, which also shows that school principals do not use humor very effectively. The reason for this could be explained by the fact that school administration is perceived as serious work, and the official dimension is more evident, as stated in the studies by Yirci et al. (2016). It should be noted that there is an understanding that humor negatively affects the seriousness of school administration and people's attitudes. In studies conducted outside of Cyprus, it was found that the positive employment of humor by school administrators was effective in the struggle to combat burnout of teachers (Ho, 2016). On the other hand, it has been argued that negative humor characteristics of school administrators negatively affect teachers in terms of their emotions and performance (Mehdinezhad and Sarooni, 2016).

When the scores obtained by the teachers in regard to the evaluation of organizational health are examined, the arithmetic mean score for the “impact of the principal” dimension is “usually.” The effectiveness of the principal in establishing a school's organizational health has once again emerged in this research. Therefore, the effect of the school principal should be taken into consideration in all aspects, and humor behaviors should be evaluated in this context. When the other sub-dimensions of the inventory related to organizational health are examined, it can be seen that similar to the “impact of the principal” dimension, the mean values are “usually” in all sub-dimensions. Regarding the organizational health inventory, the fact that the teachers stated their opinions as “usually” in all sub-dimensions reveals that elementary schools in the territory of Northern Cyprus are evaluated as being healthy. In the studies in the literature (Arokiasamy et al., 2016; Azizi and Kamali, 2016; Özgenel and Aksu, 2020), it was underlined that hiring school administrators who have strong leadership skills, as well as a positive effect on school health, is very important for overall school success.

#### Correlation Results

When the teacher opinions in Table 3 based on the relationship between the humor behaviors of school administrators and organizational health are evaluated, it can be seen that “courtesy,” which is a dimension of organizational health, is positively affected by the “productive social” and “affirmative” humor styles of the principal. The school administrator's humorous approval of teachers' behaviors and humor production shows that they attach importance to social relations. Therefore, a positive atmosphere is created at the school, and interpersonal relations are allowed to be within the framework of courtesy. In another dimension, it was seen that the non-humorous style of the administrator negatively affected organizational health in terms of courtesy and had a negative impact on the relationships in the school environment. Similar to the results of this study, Recepoglu (2011) revealed a significant relationship between school principals' humor styles and organizational health. In his study, the organizational health scores of school principals and teachers working with productive humor were high. This result was confirmed in Vickers's (2004) study conducted in the United States.

Another noteworthy finding of the study is that the school principal's rejective humor style negatively affected institutional integrity. The rejective humor style of the school administrator can reinforce the negative impact of communication and problems that are deadlocked in the school, thus damaging the integrity of the institution. In Dinç and Cemaloglu's study (Dinç and Cemaloglu, 2018), it was found that school administrators' rejective humor style caused stress among teachers.

Furthermore, we concluded that the affirmative humor style of the school administrator positively affected organizational health in the dimensions of “incentive structure,” “resource support,” “impact of the principal,” and “academic emphasis.” In the incentive dimension of organizational health, it can be said that the supportive behaviors of the school principal can increase motivation in the school and thus create an encouraging work environment. Affirmative humor style was found to be effective in the “resource support” dimension of organizational health. This shows that the school administrator considers the demands of teachers in regard to school equipment and is interested in and willing to address these shortcomings. In terms of the “impact of the principal” dimension of organizational health, the most positive humor style is again the affirmative humor style. This situation shows how effective the humor behavior of the principal can be in terms of the health of the organization. Otrar and Findikli (2014) emphasized that school administrators who adopt a positive style have a high level of life satisfaction, and they demonstrated how important humor is in this regard. A similar situation is also observed in the “academic emphasis” dimension of organizational health. It is clear that the affirmative humor style supports academic achievement. Altinkurt and Yilmaz (2011) stated that the use of effective humor in the communication process will allow decisions to be made that affect students. This situation is believed to support students' academic success. Finally, Hauseman (2020) mentions the importance of managing administrators' emotions for effective school management. The findings of this paper agree with Hauseman's (2020) study. School administrators can, first, affect teachers positively by effectively managing the emotions of people in the school. In the literature, studies show that school administrators with positive humorous characteristics increase the motivation levels of teachers in the schools where they work (Recepoglu et al., 2011). In addition, studies reveal that the humorous characteristics of school administrators can directly affect students positively; in other words, they can increase students' academic success and performance at school (Lusignolo, 2010).

Contrary to the researchers' expectations, it was concluded that the “productive social humor style” did not affect institutional integrity. This result suggests that the school administrators' ability to produce humor and their strength in social relations do not affect the school's integrity as an institution. In Akyürek's (2019) study, similar to this paper, it was revealed that institutional integrity, which is considered part of the organizational health of the institution, was the dimension least affected by social relations. This may be due to the fact that several elements are critical for ensuring the integrity of an institution. Even when a school administrator uses humor effectively and has effective leadership skills, this may not be sufficient in ensuring institutional integrity.

Another finding of the study was that the cynical humor style of the principal negatively affected the “courtesy” and “institutional integrity” dimensions in the context of organizational health. It is unsurprising that a cynical approach will not be welcomed by teachers. Naturally, it is inevitable that a cynical humor style will have a negative impact on courtesy and institutional integrity. Zengin (2018) also found that school administrators' adoption of cynical humor increased the likelihood that teachers remain silent at school.

When the school administrators' humor styles were examined in the moral dimension of organizational health, the rejective humor style was found to be more effective than the other humor styles. A principal's rejection of humor negatively affects school morale.

#### Regression Results

When Table 4, which presents the regression results of the study, is examined, we find that the different humor styles of the school administrators affect organizational health. Specifically, the cynical, productive social, and affirmative humor styles significantly predicted organizational health. Cynical humor styles of school principals had a negative effect, while productive social and affirmative humor styles had positive effects. On the other hand, the rejective and humorous styles were not significantly related to the health of the organization. That positive humor styles predicted the health of the organization once again reveals the impact of humor in school management.

It is a remarkable result that the cynical humor style, which is one of the negative approaches, predicts organizational health, while the rejective style does not. Cynicism has a direct effect on other people. Conflicts may arise between teachers and administrators who are faced with negative messages from the other person, and this tension is thought to have a negative impact on the health of the organization. However, in the rejective humor style, the interaction between the administrator and teacher is either negligible or broken. In an environment with little or no interaction, it can be said that the level of organizational health can remain constant.

Although limited research directly explains the relationship between humor and the organizational health of a school, the effect of humor on school management has been evaluated in many studies. Effective use of humor in school management reduces the effect of stress in school, calms tensions, reduces anxiety and fears, and helps to resolve conflicts (Williams, 1994; Altinkurt and Yilmaz, 2011; Aydug et al., 2018). Therefore, the humor characteristics of school administrators can affect many factors directly related to school health, such as stress, anxiety, fear, tension, and conflict (Morreall, 1991).

### Discussion on the Qualitative Dimension of the Study

When the qualitative findings are examined, participants' opinions about the effectiveness of humor in dealing with problems can be grouped as follows: preventing stressful and tense situations, creating a calming and intimate environment, helping to facilitate and persuade communication, and providing different perspectives. It can be said that humor has a positive effect on both the environment and the people working there. Likewise, humor facilitates communication by making the working environment in the school warmer and more friendly. Romero and Pescosolido (2008) stated that managers can use humor for motivation, conflict resolution, and to inspire employees.

There are two views on how the humor style adopted by a school principal affects organizational health. Some of the participants stated that the order and discipline of the school would be weakened, whereas others claimed that it would create a positive atmosphere and have positive effects. From this point of view, it is believed that school administrators can use humor for some teachers in the school environment and not for others. The individual differences of teachers may affect the school principal's decision to resort to humor. Consequently, as demonstrated by Gürbüz et al. (2013), it is evident that school principals can contribute to the health of the organization if they use positive humor styles.

## Conclusion

The findings presented here reveal once again the importance of emotions in school management and how essential emotional management is. It has been shown that a school administrator can manage emotions at school and thus, positively affect the health of the school as an organization. While educating school administrators or providing in-service training to school administrators who are active in the profession, focus should be placed especially on emotional management, effective use of humor, and the ways to improve school health. It is very important to carry out research on how obstacles to school health can be eliminated through effective emotional management and the use of humor. In this way, improvement can be achieved in effective school management.

## Data Availability Statement

The raw data supporting the conclusions of this article will be made available by the authors, without undue reservation.

## Ethics Statement

The studies involving human participants were reviewed and approved by the study was conducted after obtaining ethics approval from the Near East University ethics committee and survey application approval from North Cyprus Ministry of Education. All original data is stored by the authors. The patients/participants provided their written informed consent to participate in this study.

## Author Contributions

Mİ: conceptualization, validation, investigation, resources, data curation, and writing—review and editing. AG and Mİ: methodology, formal analysis, and writing—original draft preparation. AG: supervision and project administration. All authors worked and managed the processes together, read, and agreed to the published version of the manuscript.

## Conflict of Interest

The authors declare that the research was conducted in the absence of any commercial or financial relationships that could be construed as a potential conflict of interest.
